# Supplementary material for: The interactome of intact mitochondria by cross-linking mass spectrometry provides evidence for coexisting respiratory supercomplexes
Source: Mol Cell Proteomics. 2017 Dec 8;17(2):216–32. doi: 10.1074/mcp.RA117.000470 (PMC5795388; doi:10.1074/mcp.RA117.000470)
Supplement: Supplemental Data [file supp_RA117.000470_133922_0_supp_23978_fzffwf.zip › spectra_annotation/mito_spectra_annotation/478-1-4-1-7-1.pdf]

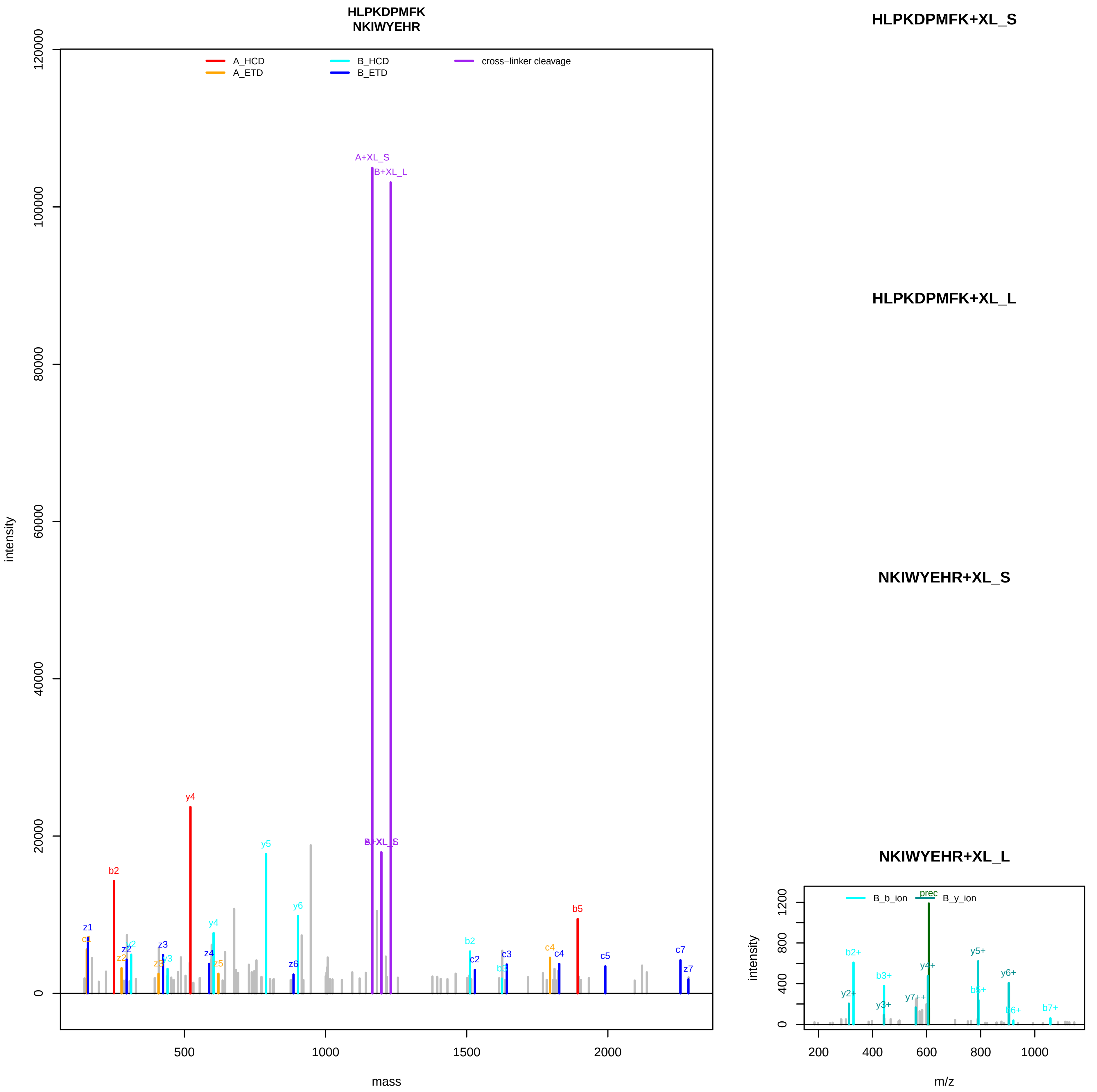

HLPKDPMFK
NKIWYEHR
A_HCD
A_ETD
B_HCD
B_ETD
cross−linker cleavage
intensity
mass
A+XL_S
B+XL_L
y4
b2
y5
y6
b5
z1
c1
z2
y2
z3
y3
z4
z5
z6
b2
c2
b3
c3
c4
c5
c7
z7
HLPKDPMFK+XL_S
HLPKDPMFK+XL_L
NKIWYEHR+XL_S
NKIWYEHR+XL_L
B_b_ion
B_y_ion
prec
b2+
b3+
y2+
y3+
y4+
y5+
y6+
y7++
b5+
b6+
b7+
intensity
m/z
